# Robotic Kidney Transplantation With Grafts With Multiple Arteries: A Single-center Experience and Outcome Analysis

**DOI:** 10.1097/TXD.0000000000001994

**Published:** 2026-08-18

**Authors:** Rossana Maffei, Haaris Kadri, Andrii Khomiak, Keith Hansen, Dor Yoeli, Wells B. LaRiviere, Shannon Lyons, Sixto Giusti, Terri Montague, James Cooper, Rashikh Choudhury, Kendra Conzen, Megan Adams, Kristin Mekeel, Peter Kennealey, Thomas Bak, Jesse D. Schold, Trevor Nydam, Thomas Pshak, Lucrezia Furian, Phillipe Abreu

**Affiliations:** 1 Department of Surgery, University of Colorado Anschutz Medical Campus, Aurora, CO.; 2 Department of Surgery, Oncology, and Gastroenterology, University Hospital of Padova, Padova, Italy.; 3 School of Medicine, University of Colorado Anschutz Medical Campus, Aurora, CO.; 4 Department of Medicine, University of Colorado Anschutz Medical Campus, Aurora, CO.

## Abstract

**Background.:**

Although robotic kidney transplantation (RKT) is an emerging minimally invasive surgical technique, graft anatomical variations such as multiple renal arteries (MRAs) remain a perceived limitation to its broader dissemination. This study evaluates the feasibility and short-term outcomes of RKT in grafts with MRAs compared with single renal artery (SRA) grafts.

**Methods.:**

We conducted a retrospective review of RKT procedures performed at a single center from November 2021 to January 2026. Perioperative and postoperative outcomes were compared between MRA and SRA cohorts. A subanalysis was performed within the MRA group to assess the impact of single versus multiple arterial anastomoses. All RKTs were performed using the Da Vinci Xi system.

**Results.:**

In the assessed period, 126 robotic-assisted kidney transplants were performed: 94 with SRA (74.6%) and 32 with MRAs (25.4%). Across the overall cohort, 1 patient (0.8%) had their operation converted to an open procedure. Total operative time had a median duration of 257 min (interquartile range [IQR], 213.0–309.8) in the SRA cohort compared with 303 min (IQR, 269.2–355.2) in the MRA cohort (*P = *0.004). Warm ischemia time was measured at 34 min (IQR, 29.0–42.0) in the SRA group and at 40 min (IQR, 33.0–45.2) in the MRA group (*P = *0.013). Within the MRA cohort, 9 grafts (28.1%) required a single arterial anastomosis (SAA) and 23 (71.9%) required multiple arterial anastomoses (MAA). The median operative time was 270 min (IQR, 210.0–305.0; SAA) versus 313 min (IQR, 283.5–392.5; MAA, *P = *0.033). Warm ischemia time, anastomosis time, blood loss, length of stay, delayed graft function, serum creatinine at 1 mo, and graft survival did not differ between SAA and MAA cohorts.

**Conclusions.:**

RKT using grafts with MRAs is technically feasible and yields perioperative and short-term outcomes comparable with those of SRA grafts. The presence of MRAs should not be considered a contraindication to robotic transplantation.

## INTRODUCTION

Kidney transplantation (KT) is considered the gold-standard treatment for end-stage renal disease, offering improved survival and a better quality of life when compared with dialysis, which is still the most common treatment option primarily due to the limited availability of donor organs in the United States and persistent disparities in access to KT.^1^ Although open KT (OKT) remains the most common procedure, robotic KT (RKT) has been considered a promising technique since 2010 when the first robotic-assisted technique was described by Giulianotti et al^2^ at the University of Chicago. Since the later descriptions of the first completely robotic cases, interest in RKT has increased, and multiple efforts have been made to expand knowledge of this procedure and its indications.

Anatomical variations in either the graft or the recipient can pose technical challenges for the surgeon during the transplant, potentially impacting the success of postoperative outcomes.^3^ Initially, grafts with a single renal artery (SRA) were considered ideal for transplantation, mostly for the reduced surgical effort and operative time.^4^ However, due to the ongoing organ shortage, grafts with multiple renal arteries (MRAs) have increasingly been accepted for transplant procedures.^5^

Kidneys with accessory arterial branches have raised significant concerns in transplantation due to an elevated risk of postoperative vascular and urological complications, such as higher incidences of vascular thrombosis, acute tubular and ureteral necrosis, impaired graft function, and prolonged cold ischemia time, attributed to the increased technical demands during back-table preparation.^5,6^ Additionally, the longer intraoperative warm ischemia time and the complexity of arterial anastomosis have further discouraged surgeons from selecting these grafts for transplantation.^3,4^

A 2020 study by Nataraj et al^7^ has previously addressed the feasibility of RKT in grafts with multiple vessels (GMVs), showing that the robotic approach does not compromise patient safety or short-term outcomes compared with OKT. Previous observational studies on OKT have indicated that grafts with MRAs have no impact on the risk of vascular injury and biopsy-proven acute tubular necrosis.^8^ There is little literature assessing comparative postoperative outcomes in patients who underwent RKT with the utilization of grafts with multiple versus single anastomoses or renal arteries. One European study highlighted no additional complication burden in patients undergoing RKT with GMVs.^9^ The work presented here is the experience of RKT with grafts with MRAs and those with multiple anastomoses (MAAs) at a single American academic transplant center. This study seeks to expand the current literature on multivessel grafts and to assess differences in complication burden in light of increased technical complexity.

## MATERIALS AND METHODS

### Patient Selection

At the University of Colorado Hospital (Aurora, CO), candidates for RKT underwent standardized preoperative evaluation protocols consistent with those used for OKT. These assessments included comprehensive surgical evaluation, review of comorbid conditions and renal function, vascular imaging, and multidisciplinary case discussion. Patient allocation to RKT versus OKT was determined primarily by the availability of the robotic system, without consideration of age, sex, or race/ethnicity. Initially, RKT was limited to elective living donor transplants; however, with the increasing institutional experience, indications were expanded to include deceased donor procedures, particularly for patients with elevated body mass index (BMI), and eventually to a broader patient population.^9^

### Surgical Technique

Vascular reconstruction of the graft vessels and preparation for transplantation were performed on the back table while the graft was preserved in ice-cold solution. Notably, in any case with multiple renal veins, reconstruction to 1 venous anastomosis was made. Preventive hemostasis was achieved using the LigaSure small jaw sealer (Medtronic, Dublin, Ireland). Gastrointestinal anastomosis staplers (Medtronic, Minneapolis, MN) were used to extend the renal vein, and 6-0 Prolene (Johnson and Johnson, New Brunswick, NJ) sutures were used for manual vascular reconstruction. Each graft was then wrapped in a QuikClot Z-Fold (Teleflex Medical, Wayne, PA) hemostatic ice jacket before being introduced into the recipient’s abdomen.

**FIGURE 1.**
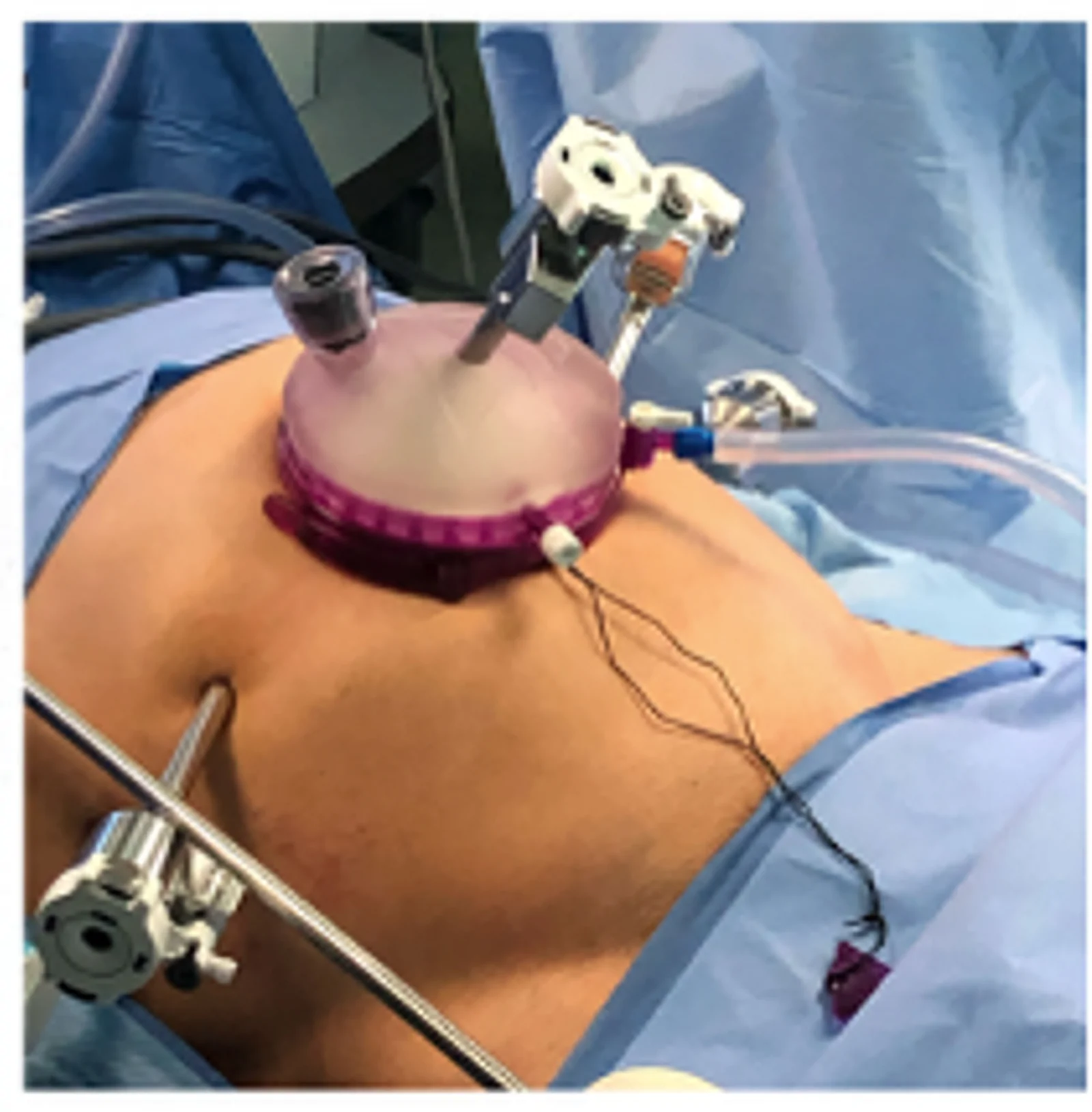
Image of port placement.

All RKTs were performed using the Da Vinci Xi robotic system (Intuitive Surgical Inc, Sunnyvale, CA). A Pfannenstiel incision was used for nonobese patients, whereas a midline incision was preferred in obese patients. A GelPOINT device (Applied Medical, Rancho Santa Margarita, CA) was placed to facilitate graft introduction. After this, additional robotic trocars were placed, as per Figure 1, and the robot was docked between the patient’s legs. A retroperitoneal pocket was created for graft placement. The external iliac vessels on the implant side were exposed, and the bladder was mobilized by developing the space of Retzius. The external iliac vein was clamped, followed by venotomy using robotic scissors. The vein was flushed with heparinized saline, and the venous anastomosis was completed using a running suture technique with either No. CV6 TTc-13 Gore-Tex sutures (Gore Medical, Newark, DE) for the initial cases or No. 5-0 CV-1 PRONOVA sutures (Johnson and Johnson MedTech, New Brunswick, NJ) for more recent cases, with a single needle cut to 16 cm. After releasing the vein clamp, the external iliac artery was clamped, and an arteriotomy was created using robotic scissors after lifting the arterial wall with a full-thickness stitch. The artery was flushed with heparinized saline, and the arterial anastomosis was also performed using a running suture technique with either No. CV6 TTc-9 Gore-Tex sutures (initial cases) or No. 6-0 RB-2 PRONOVA sutures (recent cases), with a single needle cut to 14 cm. Once vascular anastomoses were completed, robotic bulldog clamps were removed, the graft was reperfused, and hemostasis was achieved using Surgicel Fibrillar (Johnson & Johnson, New Brunswick, NJ). Renal perfusion was confirmed using indocyanine green (IC-Green, Diagnostic Green, Munich, Germany) angiography and intraoperative ultrasound. After confirming reperfusion, a nonrefluxing ureteral reimplantation was performed on the anterolateral wall of the bladder using the Lich-Gregoir technique. Anastomosis was performed in a running manner with No. 5-0 RB-1 PDS sutures (Johnson and Johnson, New Brunswick, NJ), using a single needle cut to 8 cm or a double needle cut to 16 cm, depending on the surgeon’s preference. The bladder was filled with methylene blue and normal saline with antibiotics to ensure that there was no leaking of urine into the pelvis. The Lich-Gregoir antireflux valve, as described in prior literature,^10^ was performed with No. 4-0 RB-2 STRATAFIX sutures (Johnson and Johnson, New Brunswick, NJ) in a running manner. The kidney was then positioned in the retroperitoneal space, and the peritoneum was reapproximated with either robotic Hem-o-Lok clips (Teleflex Inc, Wayne, PA) or No. 2-0 CT1 STRATAFIX sutures. Finally, the robot was undocked, and trocar sites were closed per standard surgical protocol.

A more detailed description of the used surgical technique has been published.^9^

### Data Collection and Statistical Analysis

Data for all RKT cases were extracted from the institutional electronic medical record system (EPIC Systems, Verona, WI). The analysis included procedures performed between November 2021 and January 2026. Demographic variables collected included patient age, sex, race/ethnicity, BMI, and medical comorbidities. Intraoperative data included total operative time, warm and cold ischemia times, anastomosis duration, estimated blood loss, and any conversions to open surgery. The time of reperfusion was calculated on the basis of the time at which arterial clamps were removed. Although this was always after the first anastomosis in cases with only 1 renal artery anastomosis, this was not necessarily the case in those with MAAs. Donor-related variables included donor type (living or deceased) and any anatomical variations in the renal vessels or ureter that were extracted from the electronic medical record. Particular attention was paid to the number of renal arteries and whether arterial reconstruction was required on the back table.

Postoperative outcomes included length of hospital stay, incidence and types of complications, delayed graft function (DGF), number of days on dialysis in cases of DGF, rates of 30-d readmissions and reoperations, and creatinine value at 1 mo posttransplant. DGF was defined as needing dialysis within the first week posttransplant. Both overall and cumulative graft and patient survival were compared.

All analyses were performed using R version 4.4.2 (R Foundation for Statistical Computing, Vienna, Austria). Continuous variables were compared using the Mann-Whitney *U* test, and categorical variables were compared using the chi-square or Fisher exact test as appropriate, based on sample size. Cumulative graft and patient survival were assessed using Kaplan-Meier analyses. A *P* value of <0.05 was considered significant.

This study was approved by the Colorado Multiple Institutional Review Board (protocol No. 24-2039) with a waiver of informed consent.

## RESULTS

### Baseline and Demographic Details

Among the 126 RKT procedures performed between November 2021 and January 2026, 32 involved grafts with MRA. The median age of recipients with SRA grafts was 52 y (interquartile range [IQR], 44–59.8), whereas those receiving MRA grafts had a median age of 48 y (IQR, 37–58.2; *P = *0.12). A majority of recipients in both cohorts were men: 59 (62.8%) in the SRA group and 17 (53.1%) in the MRA group (*P = *0.45), and most identified as White: 59 (62.7%) for SRA and 19 (59.3%) for MRA (*P* = 0.72). The median BMI at transplant was 35.3 kg/m^2^ (IQR, 29.3–39.4) for the SRA group and 34.5 kg/m^2^ (IQR, 30.7–39.9) for the MRA group (*P* = 0.88). Recipient, donor, and graft characteristics for both cohorts are summarized in Table 1.

**TABLE 1.** Recipient and graft characteristics for patients undergoing robotic kidney transplant with single vs multiple renal arteries

Characteristic	Single renal artery (N = 94)	Multiple renal arteries (N = 32)	*P*
Year, n (%)			0.883
2021	2 (2.2)	0 (0.0)	
2022	9 (9.8)	3 (9.4)	
2023	9 (9.8)	1 (3.1)	
2024	23 (25.0)	9 (28.1)	
2025	44 (47.8)	18 (56.2)	
2026	5 (5.4)	1 (3.1)	
Age, y, median (IQR)	52 [44.0–59.8]	48 [37.0–58.2]	0.116
Male sex, n (%)	59 (62.8)	17 (53.1)	0.451
Race/ethnicity, n (%)			0.722
Black	9 (9.57)	4 (12.5)	
Hispanic/Latino	26 (27.6)	9 (28.1)	
White, non-Hispanic/Latino	59 (62.7)	19 (59.3)	
BMI at transplant, kg/m^2^, median (IQR)	35.3 [29.3–39.4]	34.5 [30.7–39.9]	0.875
Previous kidney transplant, n (%)	4 (4.3)	2 (6.2)	0.643
Pretransplant diabetes, n (%)	34 (36.2)	12 (37.5)	1.000
Most recent serum creatinine at time of transplant, median (IQR)	6.6 [4.6–8.8]	5.4 [3.9–8.2]	0.230
Right kidney graft, n (%)	29 (30.9)	11 (34.4)	0.615
Right-sided implant, n (%)	88 (93.6)	30 (93.8)	1.000
Donor type, n (%)			0.169
DBD	20 (21.3)	9 (28.1)	
DCD	27 (28.7)	13 (40.6)	
Living	47 (50.0)	10 (31.2)	

BMI, body mass index; DBD, donor after brain death; DCD, donor after circulatory death; IQR, interquartile range.

### Subanalysis by Number of Anastomoses Within the MRA Cohort

**FIGURE 2.**
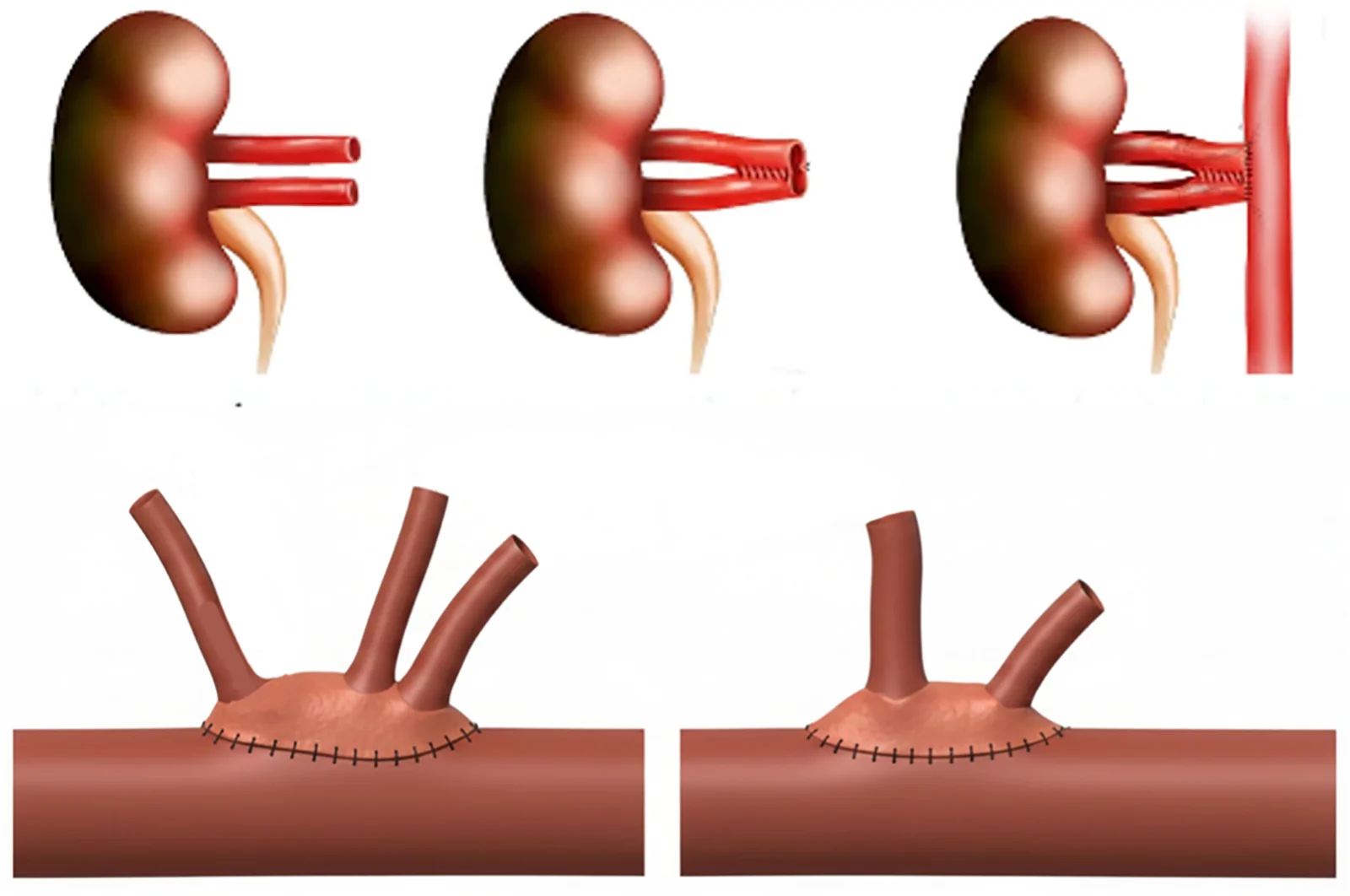
Top row: example of V-plasty between 2 renal arteries to obtain 1 final orifice for anastomosis. Bottom row: example of Carrel patch with >1 renal artery and its anastomosis.

In cases involving MRAs, surgical management was influenced by graft anatomy, surgeon experience, and recipient vessel suitability. Some grafts underwent arterial reconstruction at the back table, allowing for a single arterial anastomosis (SAA), whereas others required multiple arterial anastomoses (MAA) intraoperatively. Among the 32 MRA grafts, 9 (28%) resulted in a SAA, either due to the presence of 2 arteries on a common Carrel aortic patch or due to successful back-table reconstruction (typically V-plasty; Figure 2). These cases occurred exclusively in 2024 and 2025, reflecting the later phase of institutional experience.

**FIGURE 3.**
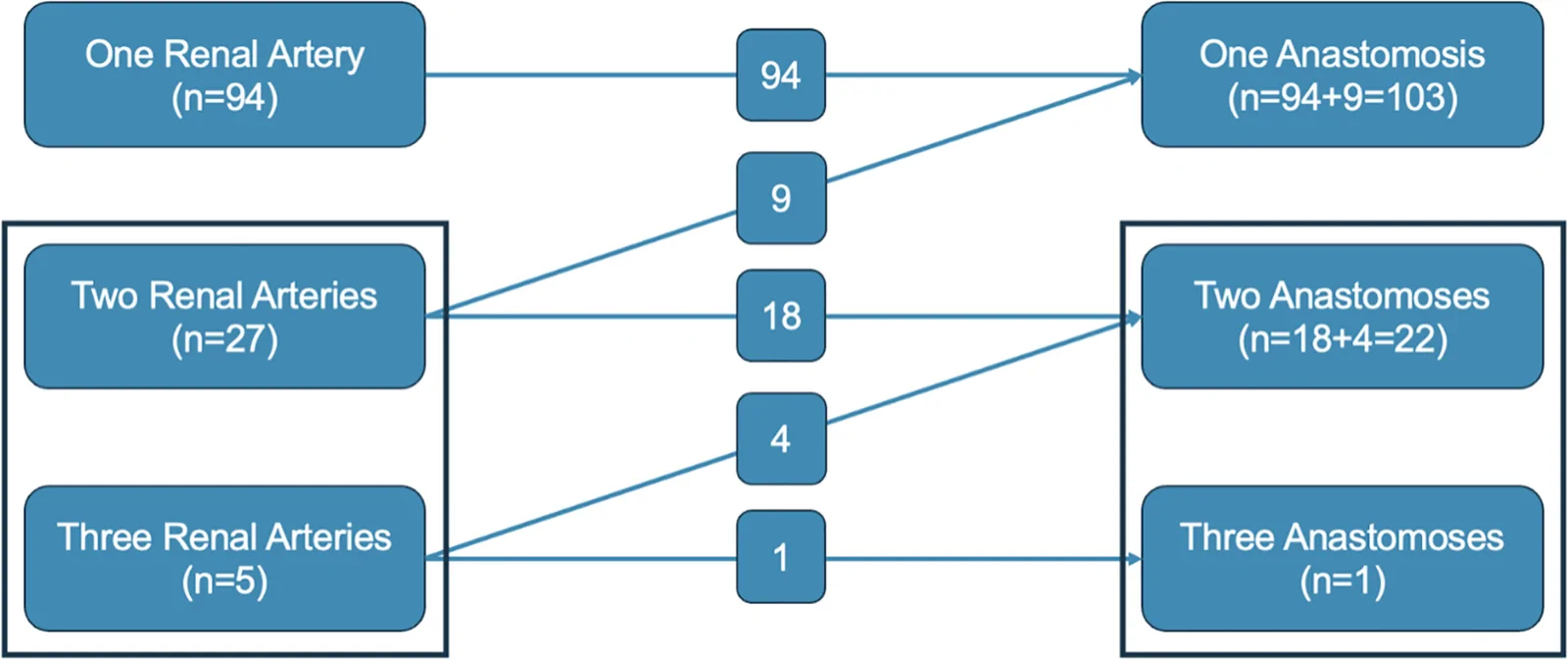
Relationship between the initial number of arteries and the final number of anastomoses.

Twenty-three cases (72%) resulted in MAA. Notably, 3 of the MAA cases were grafts with 3 arteries that required either a V-plasty or had a single aortic patch with 2 of the 3 arteries. These still required 2 anastomoses, so they were grouped within the MAA cohort for analytical clarity. A schematic representation of the relationship between the initial number of arteries and the final number of anastomoses is provided in Figure 3.

The median age of recipients with single renal anastomosis (SAA) grafts was 37 y (IQR, 36.0–59.0), whereas those receiving MAA grafts had a median age of 49 y (IQR, 42.0–57.9; *P = *0.43). The median BMI at transplant was 33.5 kg/m^2^ (IQR, 26.1–35.3) in the SAA cohort and 34.6 kg/m^2^ (IQR, 31.9–40.0) in the MAA cohort. A detailed summary of these characteristics is provided in Table 2.

**TABLE 2.** Recipient and graft characteristics for patients undergoing robotic kidney transplant with multiple renal arteries with single vs multiple arterial anastomoses

Characteristic	Single arterial anastomosis (N = 9)	Multiple arterial anastomoses (N = 23)	*P*
Year, n (%)			0.714
2021	0 (0.0)	0 (0.0)	
2022	0 (0.0)	3 (13.0)	
2023	0 (0.0)	1 (4.3)	
2024	4 (44.4)	5 (21.7)	
2025	5 (55.6)	13 (47.1)	
2026	0 (0.0)	1 (4.3)	
Age, y, median (IQR)	37.0 [36.0–59.0]	49.0 [42.0–57.0]	0.425
Male sex, n (%)	7 (77.8)	10 (43.5)	0.176
Race/ethnicity, n (%)			1.000
Black	1 (11.1)	3 (13.0)	
Hispanic/Latino	4 (44.4)	5 (21.7)	
White	4 (44.4)	15 (65.2)	
BMI at transplant, kg/m^2^, median (IQR)	33.5 [26.1–35.3]	34.6 [31.9–40.0]	0.543
Previous kidney transplant, n (%)	1 (11.1)	1 (4.3)	0.490
Pretransplant diabetes, n (%)	4 (44.4)	8 (34.8)	0.696
Most recent serum creatinine at time of transplant, median (IQR)	5.2 [4.6–8.0]	5.6 [3.6–8.4]	0.516
Right kidney graft, n (%)	7 (77.8)	4 (17.4)	**0.003**
Right-sided implant, n (%)	8 (88.9)	22 (95.7)	0.490
Donor type, n (%)			0.614
DBD	2 (22.2)	7 (30.4)	
DCD	5 (55.6)	8 (34.8)	
Living	2 (22.2)	8 (34.8)	

Bold indicates statistically significant *P* < 0.05.

DBD, donor after brain death; DCD, donor after circulatory death; IQR, interquartile range.

### Perioperative Data and Outcomes

Recipient perioperative outcomes for the SRA and MRA cohorts are summarized in Table 3. The median length of hospital stay was 3 d (IQR, 3.0–4.0) for both cohorts (*P = *0.27). The total operative time had a median duration of 257 min (IQR, 213.0–309.8) in the SRA cohort compared with 303 min (IQR, 269.2–355.2) in the MRA cohort (*P = *0.004). The median warm ischemia time was 34 min (IQR, 29.0–42.0) in the SRA group and 40 min (IQR, 33.0–45.2) in the MRA group (*P = *0.013). Conversion to open surgery was required in 1 case (1.1%) in the SRA group (due to intraoperative concerns of inadequate venous drainage), whereas no conversions were reported in the MRA cohort (*P = *1.00). Estimated intraoperative blood loss was a median of 50 mL (IQR, 10.0–100.0) in the SRA group and 60 mL (IQR, 20.0–100.0) in the MRA group (*P = *0.18).

**TABLE 3.** Outcomes for patients undergoing robotic kidney transplant with single vs multiple renal arteries

Characteristic	Single renal artery (N = 94)	Multiple renal arteries (N = 32)	*P*
Operative time, min, median (IQR)	257.0 [213.0–309.8]	303.0 [269.2–355.2]	**0.004**
Warm ischemic time, min, median (IQR)	34.0 [29.0–42.0]	40.0 [33.0–45.2]	0.013
Estimated blood loss, mL, median (IQR)	50.0 [10.0–100.0]	60.0 [20.0–100.0]	0.179
Conversion to open, n (%)	1 (1.1)	0 (0.0)	1.000
Length of stay posttransplant, d, median (IQR)	3.0 [3.0–4.0]	3.0 [3.0–4.0]	0.269
Reoperation within 30 d, n (%)	4 (4.3)	2 (6.2)	0.643
Readmission within 30 d, n (%)	17 (18.1)	6 (18.8)	1.000
Surgical site infection, n (%)	1 (1.1)	1 (3.1)	0.445
Seroma, n (%)	4 (4.3)	3 (9.4)	0.369
Hematoma, n (%)	5 (5.3)	2 (6.2)	1.000
Urine leak, n (%)	2 (2.1)	0 (0.0)	1.000
Ureteral stricture, n (%)	0 (0.0)	1 (3.1)	0.254
Incisional hernia, n (%)	2 (2.1)	2 (6.2)	0.267
Lymphocele, n (%)	0 (0.0)	2 (6.2)	0.063
Delayed graft function, n (%)	25 (26.6)	5 (16.1)	0.333
Days on dialysis posttransplant,^*a*^ median (IQR)	14.0 [7.0–18.0]	12.0 [8.0–17.0]	0.737
Serum creatinine at discharge, median (IQR)	2.5 [1.6–5.3]	2.2 [1.6–3.9]	0.252
Serum creatinine at 1 mo posttransplant, median (IQR)	1.6 [1.3–2.0]	1.5 [1.2–1.9]	0.308
Length of follow-up, d, median (IQR)	177.5 [83.5–359.0]	133.0 [82.8–358.8]	0.646

Bold indicates statistically significant *P* < 0.05.

IQR, interquartile range.

^*a*^Variable only considered for patients with delayed graft function.

The 30-d readmission rate was 18.1% in the SRA group and 18.8% in the MRA group (*P = *1.00). Reoperation within 30 d occurred in 4 cases (4.3%) in the SRA cohort compared with 2 in the MRA group (6.2%; *P = *0.643). Reoperations in the SRA cohort included 1 wound hematoma evacuation, 1 incisional hernia repair, 1 exploratory laparoscopy with kidney biopsy, and 1 robotic ureteral reimplantation. In the MRA group, the 2 reoperations were both performed for hematoma washout.

Postoperative complications were observed in both cohorts. In the SRA group, these included 1 case of surgical site infection (1.1%), 4 seromas (4.3%), 5 hematomas (5.3%; 1 requiring reoperation), and 2 urinary leaks (2.1%), of which 1 was managed surgically and the other via interventional radiology nephrostomy, and 2 cases of incisional hernias (2.1%, 1 requiring reoperation within 30 d). In the MRA cohort, complications included 1 surgical site infection (3.1%), 3 seromas (9.4%), 2 hematomas (6.2%, both requiring reoperation), 1 ureteral stricture (3.1%, managed endoscopically with stent placement), 2 incisional hernias (6.2%), 2 lymphoceles (6.2%, 1 requiring drainage), and 1 case of ureteral stricture (3.1%, requiring reoperation). All seromas in both groups were managed conservatively without surgical intervention.

**FIGURE 4.**
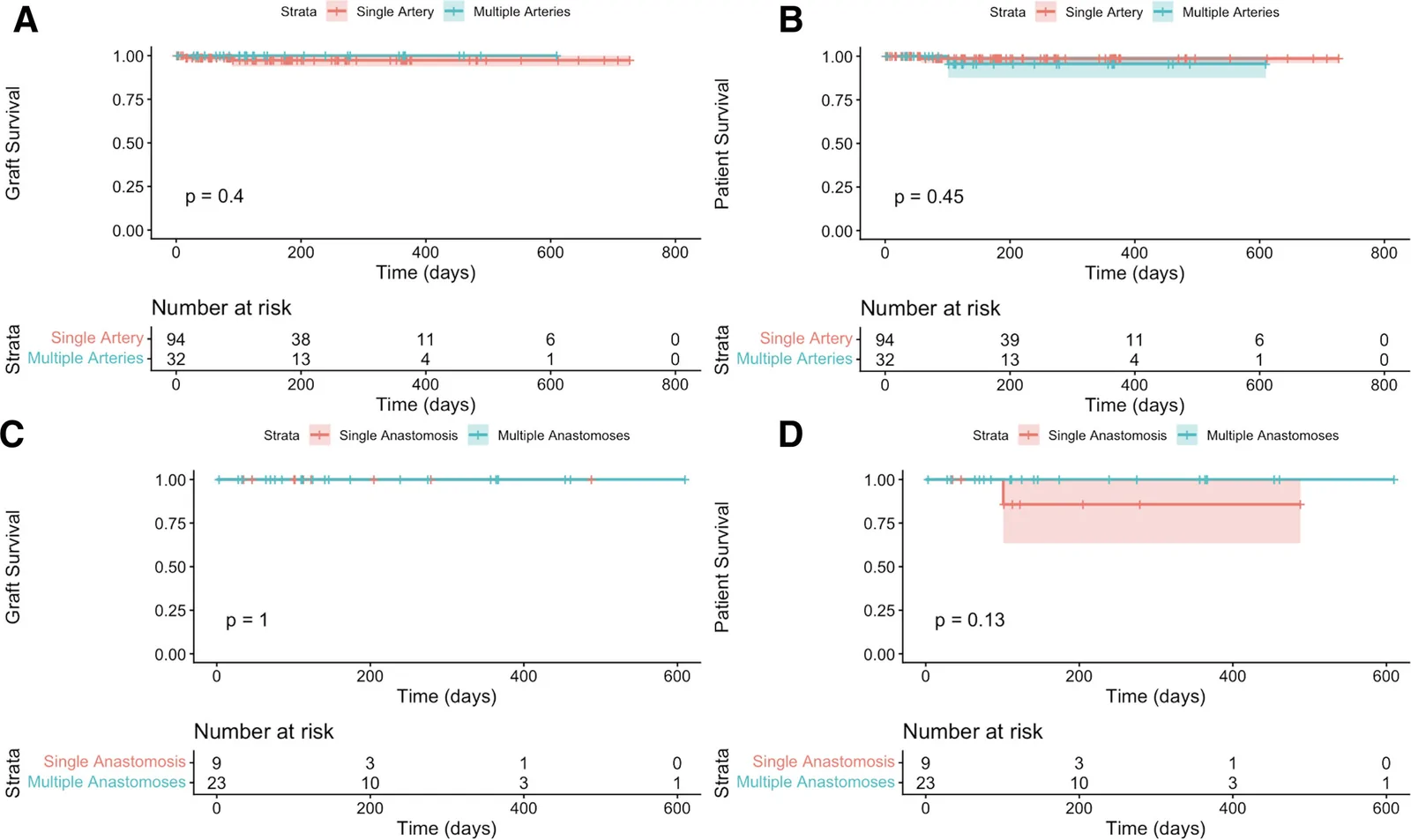
Comparison of graft (A) and patient (B) survival between single and multiple artery graft transplants, and graft (C) and patient (D) survival in single and multiple anastomosis graft transplants in those with multiple renal arteries.

DGF occurred in 26.6% versus 16.1% of patients in the SRA and MRA cohorts, respectively (*P* = 0.35). The median time on dialysis for these patients did not differ significantly between cohorts (*P* = 0.74). No other postoperative outcomes differed significantly. Serum creatinine at 1 mo posttransplant was compared, with values of 1.6 mg/dL (IQR, 1.3–2.0) in the SRA cohort and 1.5 mg/dL (IQR, 1.2–1.9) in the MRA cohort (*P = *0.308). At comparable follow-up between SRA and MRA cohorts (median 177.5 versus 133.0 d, *P *= 0.646), graft and patient survival did not differ (Figure 4A and B).

### Subanalysis Based on Number of Anastomoses Within the MRA Cohort

A subanalysis was conducted within the MRA cohort to compare intraoperative and postoperative outcomes between cases requiring a SAA (n = 9) and those requiring MAA (n = 23).

Peri- and postoperative outcomes between the SAA and MAA cohorts are compared in Table 4. The median postoperative length of stay was similar between the 2 cohorts (4.0 versus 3.0 d, *P = *0.21). The total operative time had a median duration of 270.0 min (IQR, 210.0–305.0) in the SAA group, which was significantly <313.0 min (IQR, 283.5–392.5) in the MAA group (*P = *0.033). Warm ischemia time was measured at 40 min (IQR, 33.0–40.0) in the SAA group and 41 min (IQR, 36.5–47.0) in the MAA group (*P = *0.38). Estimated intraoperative blood loss was a median of 20 mL (IQR, 10.0–100.0) in the SAA group and 100 mL (IQR, 50.0–100.0) in the MAA group (*P = *0.08). Additionally, serum creatinine at discharge was 2.1 mg/dL (IQR, 1.8–4.6) in the former cohort and 2.4 mg/dL (IQR, 1.4–3.4) in the latter cohort (*P = *0.451). Finally, 1 patient (11.1%) in the SAA cohort developed DGF compared with 4 patients (18.2%) in the MAA cohort. No postoperative complication rates differed between SAA and MAA cohorts (Table 4). Cumulative graft and patient survival did not differ significantly between cohorts at follow-up (Figure 4C and D).

**TABLE 4.** Outcomes for patients undergoing robotic kidney transplant with multiple renal arteries with single vs multiple arterial anastomoses

Characteristic	Single arterial anastomosis (N = 9)	Multiple arterial anastomoses (N = 23)	*P*
Operative time, min, median (IQR)	270.0 [210.0–305.0]	313.0 [283.5–392.5]	**0.033**
Warm ischemic time, min, median (IQR)	40.0 [33.0–40.0]	41.0 [36.5–47.0]	0.378
Estimated blood loss, mL, median (IQR)	20.0 [10.0–100.0]	100.0 [50.0–100.0]	0.078
Conversion to open, n (%)	1 (1.0)	0 (0.0)	1.000
Length of stay posttransplant, d, median (IQR)	4.0 [3.0–4.0]	3.0 [3.0–3.5]	0.209
Reoperation within 30 d, n (%)	1 (11.1)	1 (4.3)	0.490
Readmission within 30 d, n (%)	2 (22.2)	4 (17.4)	1.000
Surgical site infection, n (%)	0 (0.0)	1 (4.3)	1.000
Seroma, n (%)	0 (0.0)	3 (13.0)	0.541
Hematoma, n (%)	0 (0.0)	2 (8.7)	0.610
Urine leak, n (%)	0 (0.0)	0 (0.0)	1.000
Ureteral stricture, n (%)	0 (0.0)	1 (4.3)	1.000
Incisional hernia, n (%)	1 (11.1)	1 (4.3)	0.490
Lymphocele, n (%)	0 (0.0)	2 (8.7)	1.000
Delayed graft function, n (%)	1 (11.1)	4 (18.2)	1.000
Days on dialysis posttransplant,^*a*^ median (IQR)	17.0 [17.0–17.0]	10.0 [7.0–18.5]	0.480
Serum creatinine at discharge, median (IQR)	2.1 [1.8–4.6]	2.4 [1.4–3.4]	0.451
Serum creatinine at 1 mo posttransplant, median (IQR)	1.3 [1.1–1.8]	1.6 [1.4–1.9]	0.170
Length of follow-up, d, median (IQR)	113.0 [101.0–205.0]	146.0 [80.5–364.0]	0.516

Bold indicates statistically significant *P* < 0.05.

IQR, interquartile range.

^*a*^Variable only considered for patients with delayed graft function.

## DISCUSSION

These analyses highlight that neither MRAs nor MRA anastomoses convey poorer outcomes in the perioperative setting after RKT. The increase in MRA cases between 2021 and 2026 demonstrates a trend that likely reflects both the progressive expansion of robotic platform availability and the increasing technical proficiency of the surgical team. In this analysis, we found no significant differences in postoperative complications when comparing SRA and MRA cohorts, as well as in the SAA and MAA cohorts. Other outcomes of importance, such as rates of DGF and graft or patient survival, did not differ significantly, consistent with prior literature.^11,12^

At our center, the preference is to perform MRA anastomoses with grafts that have MRAs for 2 reasons, as outlined here. First, if a clot occurs in a small additional renal artery, then keeping the anastomoses separate allows for retention of kidney function, as the main renal artery would still be patent. If the arteries were joined during back-table preparation, then a clot in 1 artery would compromise the entire graft. Second, positioning the kidney in the retroperitoneal pocket via a robotic approach is easier with 2 separate anastomoses. If back-table preparation led to 1 anastomosis from multiple arteries, then the 3-dimensional relationships of the vessels may lead to functional occlusion and difficult graft positioning. Hence, we reconstruct vessels on the back table only when they are too close to each other, and it is feasible to do a side-to-side pantaloon technique. An end-to-side anastomosis was performed only in 1 case as a last resort. Whenever the arteries are far apart enough to allow multiple openings in the iliac artery, that is the preference at our center.

In this analysis, we found that the MRA cohort had longer operative and warm ischemia times by 46 and 6 min, respectively. The MAA cohort had longer operative and warm ischemia times by 43 and 1 min, respectively. This lengthening of operative time without as dramatic an increase in warm ischemia time is interesting but explainable. In our experience, the additional operative time is conferred by the more complex dissection of the iliac vessels and the more extensive preparation of the iliac artery, which allows wider clamping for MAAs. Additionally, portions of the operation, such as assessment of hemostasis and graft positioning, take longer. These changes largely affect the total operative time, with the warm ischemia time being less affected.

Siena et al^13^ reported outcomes specifically from 148 RKT cases, of which 21 (14.2%) involved GMVs. In this study, all GMV grafts underwent back-table vascular reconstruction, allowing for a SAA during implantation. Although the GMV group exhibited longer operative times, consistent with our findings, it also demonstrated significantly longer cold ischemia and anastomosis times. In contrast, our study deliberately included grafts with multiple arteries, which were implanted with either single or MAA, depending on intraoperative considerations. Although total operative time, warm ischemia time, and anastomosis time were increased in the MRA cohort as well as total operative time in its MAA subgroup, no other statistically significant differences were observed in perioperative parameters, complication rate, or type.

Similarly, Kishore et al^14^ analyzed 18 RKT procedures performed with GMV grafts from living donors, all conducted by a single surgeon at a single institution. Outcomes were retrospectively compared with RKT using single-vessel grafts and OKT with GMVs, revealing no significant differences in graft function or perioperative outcomes among the groups.^14^ Unlike their work, our analysis included both living and deceased donor grafts. In our study, donor type did not differ significantly in the SRA versus MRA comparison, but more grafts that had 1 arterial anastomosis came from deceased donors.

A major strength of this study is the uniform application of robotic technique across all 126 transplant procedures, irrespective of the number of renal arteries or arterial anastomoses required. Additionally, the inclusion of a heterogeneous donor population, comprising 69 deceased donors (54.7% of cases), enhances the clinical relevance and novelty of our findings. Unlike prior reports limited to living donors or standardized vascular reconstructions, this study reflects real-world surgical variability and intraoperative decision-making, consistent with the evolution of transplantation. Of interest, we found that many of the SRA grafts were from living donors compared with the MRA cohort, whereas the opposite was observed in the anastomoses-level analysis. Although neither of these differences was significant, it should be noted that the larger proportion of the MAAs cohort being from living donors may be due to the inability to create a Carrel patch with living donors. Although rates of DGF did not differ significantly between cohorts, it is possible that differences that were observed may be secondary to factors that have been shown to affect postoperative renal function, such as donor type. Although this is difficult to assess given the modest sample size in our cohort, future work may assess how donor type affects outcomes in the context of specific donor anatomy. The effect of donor anatomy on rates of DGF has been studied,^11,12^ with but it is unclear how this may augment or affect clinical decision-making at this time.

Nevertheless, several limitations must be acknowledged. The relatively small sample size and single-center design limit the generalizability of the results. Moreover, the analysis was confined to short-term perioperative outcomes, and long-term graft and patient survival data are not yet available for all patients. Other clinically relevant variables, such as arterial flow, were not compared in this work, although this would be an interesting topic for a future prospective trial. Despite these limitations, to our knowledge, this is the first American study evaluating RKT outcomes in both single and MRA grafts performed entirely with a robotic approach. These findings contribute meaningfully to the growing body of evidence supporting robotic transplantation and demonstrate that the presence of MRAs does not adversely affect short-term outcomes. Future research should compare outcomes in GMVs transplanted via open and RKT with more robust sample sizes. Our findings suggest that the requirement for MAA should not be a deterrent to the use of robotic-assisted KT. Perioperative and short-term outcomes were comparable between single and multiple artery graft recipients.
